# *Cryptosporidium* and *Giardia* prevalence amongst lemurs, humans, domestic animals and black rats in Tsinjoarivo, Madagascar

**DOI:** 10.1016/j.heliyon.2020.e05604

**Published:** 2020-11-30

**Authors:** Laurie A. Spencer, Mitchell T. Irwin

**Affiliations:** a Department of Biological Sciences, Northern Illinois University, 1425 W. Lincoln Highway, Montgomery Hall 349, DeKalb, IL 60115, USA; b Department of Anthropology, Northern Illinois University, 1425 W. Lincoln Highway, Stevens Building 275, DeKalb, IL 60115, USA

**Keywords:** Microbiology, Zoology, Public health, Infectious disease, *Cryptosporidium*, *Giardia*, Lemurs, Humans, Domestic animals, Black rats

## Abstract

Few studies have measured the prevalence of *Cryptosporidium* sp. and *Giardia* sp. infections in Madagascar. This project provides baseline data of these pathogens in humans and other mammals in Tsinjoarivo. Fecal samples were collected May–July 2014 from lemurs (*Propithecus diadema* and *Hapalemur griseus*), humans, domestic animals (cattle, pigs and dogs), and black rats (*Rattus rattus*). Samples were analyzed utilizing immunofluorescence assay. No lemurs were positive for either parasite. *Cryptosporidium* sp. was found in humans (10%), cattle (20%), pigs (20%), dogs (15%) and rats (38%), and *Giardia* sp. was found in humans (10%), pigs (40%), dogs (29%) and rats (53%). Coinfections were noted in humans (6%), pigs (20%), dogs (15%) and rats (33%). All human subjects reported daily contact with domestic animals and rats, and all infected humans were ≤13 years old. Human population growth and increasing human-wildlife encounters make it critical to understand the potential for zoonotic pathogen transmission.

## 1 Introduction

Improved understanding of pathogen transmission pathways amongst humans, domestic animals and wildlife is crucial for maintaining the health of all species and for conservation of threatened wildlife [1].

*Cryptosporidium* sp. and *Giardia* sp. are ubiquitous enteric protozoan pathogens that infect humans, domestic animals and wildlife worldwide [2]. *Cryptosporidium* sp. oocysts and *Giardia* sp. cysts are transmitted by the fecal-oral route, largely through contaminated water and food, but can also spread via direct contact with an infected individual [3]. Poor hygiene and sanitation conditions and contact with domestic animals are risk factors for human infection [4, 5]. These factors along with high occurrence of untreated drinking water and open defecation by humans and domestic animals puts the inhabitants of Madagascar at risk for exposure to *Cryptosporidium* sp. and *Giardia* sp [6].

*Cryptosporidium* sp. has been found in a few lemur species [7, 8, 9], with no reports of *Giardia* sp. to date. Both parasites have been documented in Malagasy children [10, 11]. No data were found on *Cryptosporidium* sp. or *Giardia* sp. infections in domestic animals in Madagascar. The overall health burden from these infections in Madagascar remains under-studied.

This study was designed to address the paucity of data on *Cryptosporidium* sp. and *Giardia* sp. infections in Madagascar utilizing a One Health approach. The objectives were to (i) measure the prevalence of *Cryptosporidium* sp. and *Giardia* sp. in sympatric lemurs, humans, domestic animals and commensal rats; (ii) assess risk factors for infection; (iii) test the hypothesis that habitat disturbance increases the prevalence of both parasites in lemurs; (iv) measure the prevalence of coinfections.

## 2 Materials and methods

Tsinjoarivo, Madagascar is located 80 km SSE of Antananarivo, atop the escarpment dividing Madagascar's central plateau from the eastern lowlands (Supplementary Data, Figure S1). This region contains mid-altitude rain forest [12] within the new Tsinjoarivo-Ambalaomby protected area. This study was conducted at two field sites: Mahatsinjo and Ankadivory. Mahatsinjo (19°40.940 S, 47°45.460 E) is about 1590 m above sea level and is comprised of forest fragments (range 1–227 ha) where human and nonhuman primate populations live side by side [13]. Mahatsinjo offers no access to electricity or sanitation infrastructure, hygiene is poor and open defecation by humans and domestic animals is the norm. Ankadivory (19°42.980 S, 47°49.293 E) is about 1345m above sea level and consists of continuous forest with higher plant species richness [13, 14]; humans are present but less common than Mahatsinjo. Distinct wet (December–March) and dry (April–November) seasons exist [14].

Use of animals in this study was approved by the Institutional Animal Care and Use Committee at Northern Illinois University (ORC #LA12-0011). Use of human subjects was approved by the Northern Illinois University Institutional Review Board (Protocol #HS14-0138). Informed consent was obtained, and the nature and possible consequences of the study was fully explained for all human subjects. Biological sample collection and laboratory analysis protocols were approved by the Northern Illinois University Institutional Biosafety Committee (ORC #S14-0006).

A total of 298 fecal samples were collected from Diademed sifaka (*Propithecus diadema*), Eastern lesser bamboo lemur (*Hapalemur griseus*), Human (*Homo sapiens*), Domestic cattle/zebu (*Bos indicus*), Domestic pig (*Sus scrofa*), Domestic dog (*Canis familiaris*), and black rat (*Rattus rattus*) (Table 1; Supplementary Data, Tables S3–S4) between May and July 2014. For each sample, an approximately 2-gram aliquot was preserved in 10% formalin solution. Lemur groups were followed, and fecal samples were collected from the ground promptly after defecation was observed. Humans living within 5 km of the forest were invited to participate in the study. Participants were asked a series of questions regarding demographics, behaviors and gastrointestinal symptoms. Participant recruitment, informed consent, survey questionnaire and debriefing scripts can be found in Supplementary Data, Appendices A-D. Human participants were also asked for permission to collect feces from their domestic mammals, and samples were collected immediately after observed defecation. For black rats (*Rattus rattus*), Sherman traps and locally sourced rat traps were set at the human participants’ homes and within the intact forest (Ankadivory). Traps were set at approximately 16:00 h and were baited with various food items: peanut butter, crunchy peanut butter Clif Bars®, dried fish, and chicken bones (highest trapping success was achieved using chicken bones). Each trap was checked the following morning at 07:00 h. The rats were released at the site of capture and fecal material was collected from inside the traps. Before reuse, each trap was cleaned and decontaminated with a 10% sodium hypochlorite solution.

**Table 1:** Quantity of fecal samples collected at Tsinjoarivo, Madagascar, per species.

Species	Total Samples	Adult – Females	Adult - Males	Adult – Sex Unknown	Immature - Females	Immature - Males
Human (*Homo sapiens*)	49	10	8	0	15	16
Black Rat (*Rattus rattus*)^1^	40	N/A	N/A	N/A	N/A	N/A
Zebu (*Bos indicus*)	41	19	14	6	0	2
Pig (*Sus scrofa*)	40	2	0	1	20	17
Dog (*Canis familiaris*)	41	8	23	1	6	3
Diademed Sifaka (*Propithecus diadema*)^2^	43	14	16	2	Sex undetermined: 11	
Eastern Bamboo Lemur (*Hapalemur griseus*)^2^	44	15	10	0	Sex undetermined: 19	
**Grand Total**	**298**					

1 Sex and age of rats was not determined during capture.

2 Due to a relatively small number of habituated individuals, individual lemurs were sampled repeatedly, whereas subjects from all other species were sampled only once.

All samples were concentrated using the Fecal Parasite Concentrator kit (Evergreen Scientific Inc. Los Angeles, California), then microscopically analyzed using a fluorescence microscope and the Merifluor *Cryptosporidium*/*Giardia* kit (Meridian Bioscience, Inc. Cincinnati, Ohio) [8, 15].

All statistical analyses were conducted in R version 3.2.2 [16]. Statistical significance was set to α = 0.05. Fisher's Exact tests were used to analyze differences in infection prevalence among host species. The association between various exposure variables (age, sex, contact with lemurs, forest entry, living with another infected person, living with an infected domestic mammal, and reporting of gastrointestinal symptoms) and the outcome of human infection were tested using Odds Ratio tests for binary variables. Additionally, the association between exposure variables (age, number of people in household, number of domestic mammals, number of cattle in household, number of pigs in household, number of dogs in household, frequency of direct contact with lemurs, and frequency of entering the forest) and the outcome of human infection were analyzed using Logistic Regression tests. For domestic mammals, the association between animal age and number of animals in household and the outcome of infection were tested using Logistic Regression. Coinfections of *Cryptosporidium* sp. and *Giardia* sp. were tested using Odds Ratio and Fisher's Exact tests.

## 3 Results

Prevalence estimates of *Cryptosporidium* sp. and *Giardia* sp. are summarized in Table 2. *Cryptosporidium* sp. was detected in 42 out of 298 fecal samples (14%). The prevalence of *Cryptosporidium* sp. was highest in rats (38%), followed by cattle (20%), pigs (20%), dogs (15%), and humans (10%). *Cryptosporidium* sp. was not detected in any lemur samples. *Giardia* sp. was detected in 54 out of 298 (18%) fecal samples. The prevalence of *Giardia* sp. was highest in rats (53%), followed by pigs (40%), dogs (29%), and humans (10%). *Giardia* sp. was not detected in lemur or cattle samples.

**Table 2:** Prevalence of *Cryptosporidium* sp. and *Giardia* sp. infections, per species.

Host	Cryptosporidium			Giardia		
	Positive/Total	Prevalence	95% Confidence Interval	Positive/Total	Prevalence	95% Confidence Interval
Human (*Homo sapiens*)	5/49	0.10	0.04–0.23	5/49	0.10	0.04–0.23
Black Rat (*Rattus rattus*)	15/40	0.38	0.23–0.54	21/40	0.53	0.36–0.68
Cattle/zebu (*Bos indicus*)	8/41	0.20	0.09–0.35	0/41	0.00	0.00–0.11
Pig (*Sus scrofa*)	8/40	0.20	0.10–0.36	16/40	0.40	0.25–0.57
Dog (*Canis familiaris*)	6/41	0.15	0.06–0.30	12/41	0.29	0.17–0.46
Diademed Sifaka (*Propithecus diadema*)	0/43	0.00	0.00–0.10	0/43	0.00	0.00–0.10
Eastern Bamboo Lemur (*Hapalemur griseus*)	0/44	0.00	0.00–0.10	0/44	0.00	0.00–0.10

None of the potential risk factors showed a statistically significant association with infection of *Cryptosporidium* sp. or *Giardia* sp. (Supplementary Data, Tables S1–S2 and Supplementary Data, Figures S2–S6). It is worth noting that all humans infected with either parasite were children ≤13 years of age. All human subjects reported daily direct contact with domestic animals and rats and daily entering into the forest. None of the infected humans had direct contact with lemurs or had an infected domestic animal in their household. Since no lemurs tested positive for either parasite, the hypothesis that increased habitat disturbance also increases infection prevalence could not be tested.

Overall, coinfections (infection with both *Cryptosporidium* sp. and *Giardia* sp.) were detected in 30 out of 298 (10%) fecal samples; these included humans, rats, pigs and dogs (Table 3; Supplementary Data, Table S5). The prevalence of coinfection was highest in rats (33%), followed by pigs (20%), dogs (15%), and humans (6%).

**Table 3:** Coinfections of *Cryptosporidium* sp. and *Giardia* sp. for humans, pigs, dogs and rats.

Host	Coinfected/Total	Prevalence	Odds Ratio	95% Confidence Interval	P
Human (*Homo sapiens*)	3/49	0.06	26.31	1.97–514.94	0.005
Pig (*Sus scrofa*)	8/40	0.20	Inf. (∞)	3.84 - Inf. (∞)	0.0002
Dog (*Canis familiaris*)	6/41	0.15	Inf. (∞)	4.10 - Inf. (∞)	0.0002
Black Rat *(Rattus rattus*)	13/40	0.33	12.81	2.15–143.87	0.001

## 4 Discussion

Both parasites were detected in humans, domestic animals and rats near the fragmented forest (Mahatsinjo). All lemurs tested negative for these parasites. This result is not consistent with other studies showing that primates living in disturbed habitats are at greater risk for *Cryptosporidium* sp. and *Giardia* sp. infection originating from humans or domestic animals [15, 17]. Domestic animals from this study were observed roaming and defecating in and near forest edges. If human and domestic animal encroachment into lemur habitat continues to increase, the lemurs of Tsinjoarivo may be at increasing risk of cross-species infection with *Cryptosporidium* sp., *Giardia* sp. or other pathogens.

This study revealed that all human infections were in children ranging from 2.5 to 13 years old. This is consistent with previous studies that identified cryptosporidiosis and giardiasis cases only in Malagasy children [10, 11].

The prevalence of *Cryptosporidium* sp. and *Giardia* sp. infections was higher in domestic animals than in humans. If cross-species transmission is occurring, this population of people may be at risk of repeated infections from direct contact with their animals. Contact with black rats may pose the greatest risk for infection since rats in this study had the highest prevalence of *Cryptosporidium* sp. (38%) and *Giardia* sp. (53%), and the highest coinfection rate (33%). Thirty-nine out of 40 the rats in this study were captured near human households and 100% of human participants reported daily direct contact with rats. Rodents are reservoirs for several zoonotic diseases responsible for significant economic and health consequences [18, 19]. Black rats have been seen within both forest fragments and in continuous forest sites at Tsinjoarivo. They have been observed climbing trees and may thereby be a threat in terms of transmitting disease to wild lemurs. The 1 rat sampled from the intact forest site (Ankadivory) was negative for both parasites. Time restraints prevented us from sampling more rats in the intact forest system.

Several study limitations were recognized. This study included relatively small sample sizes; increased sampling could increase statistical power, which is critical when infection prevalence is low (10% or less). No samples were collected during the rainy season, so effects of seasonality on infection prevalence could not be assessed. Longitudinal cohort studies involving repeated sampling of subjects would improve estimates as single samples are likely to underestimate infection prevalence [20]. The immunofluorescence method used herein allowed for parasite detection at the genus level; further molecular and phylogenetic analysis would allow for elucidation of possible cross-species transmission. Future research should include testing various water sources in conjunction with molecular characterization of parasite isolates, as this could provide insight into transmission routes. This study focused on two parasites, and additional research is needed to understand what other pathogens may be circulating in this system. A strength of this study is that the immunofluorescence detection method is highly specific and not likely to produce false positives. Additionally, all human subjects were provided with their test results as well as information about how these parasites are spread and tips for prevention (Supplementary Data, Appendix D). Results from this study can provide data to support the need for initiatives aimed at improving hygiene and water sanitation, reducing open defecation, reducing invasive rat populations and providing education on the fecal-oral route of transmission of pathogens.

## Declarations

### Author contribution statement

Laurie A. Spencer: Conceived and designed the experiments; Performed the experiments; Analyzed and interpreted the data; Contributed reagents, materials, analysis tools or data; Wrote the paper.

Mitchell T. Irwin: Analyzed and interpreted the data; Contributed reagents, materials, analysis tools or data.

### Funding statement

This work was supported by Laurie A. Spencer and the Anthropology Foundation Fund and Graduate School Travel Fund from Northern Illinois University.

### Competing interest statement

The authors declare no conflict of interest.

### Additional information

No additional information is available for this paper.

## Appendix A Supplementary data

Appendices A-D.docx A. Recruitment: Information Script B. Informed Consent Documents C. Materials: Questionnaire/Data to be collected D. Debriefing Information: Script for Human Participants with Positive (Infected) Test Results for *Cryptosporidium* sp. and/or *Giardia* sp. Appendices A-D.docx

Figure S1.docx **Figure S1:** Map of study site location – New Protected Area (NAP) Tsinjoarivo-Ambalaomby. Figure S1.docx

Figures S2-S6.docx **Figure S2:** Covariance between continuous risk factors and *Cryptosporidium* sp. infection (0=uninfected, 1=infected) in humans, showing logistic regression in blue. a) Age; b) Household size; c) Number of domestic animals in household; d) Number of cattle; e) Number of pigs; f) Number of dogs; g) Frequency of lemur contact; h) Frequency of forest entry. **Figure S3:** Covariance between continuous risk factors and *Giardia* sp. infection (0=uninfected, 1=infected) in humans, showing logistic regression in blue. a) Age; b) Household size; c) Number of domestic animals in household; d) Number of cattle; e) Number of pigs; f) Number of dogs; g) Frequency of lemur contact; h) Frequency of forest entry. **Figure S4:** Covariance between domestic mammal’s age and *Cryptosporidium* sp. infection (0=uninfected, 1=infected), showing logistic regression in blue. a) Cattle; b) Pigs; c) Dogs. **Figure S5:** Covariance between domestic mammal’s age and *Giardia* sp. infection (0=uninfected, 1=infected), showing logistic regression in blue. a) Pigs; b) Dogs. **Figure S6:** Covariance between the number of domestic mammals in the household and the likelihood of an individual mammal’s infection state (0=uninfected, 1=infected), showing logistic regression in blue. a) *Cryptosporidium* sp.; b) *Giardia* sp.; c) *Cryptosporidium* sp. and/or *Giardia* sp. Figures S2-S6.docx

Tables S1-S2.docx **Table S1:** Effects of categorical risk factors for *Cryptosporidium* sp. or *Giardia* sp. infections in humans using Odds Ratio test for binary categories. **Table S2:** Effects of continuous risk factors on *Cryptosporidium* sp. or *Giardia* sp. infections in humans using logistic regression (positive Z represents a positive relationship between the variable and likelihood of infection). Tables S1-S2.docx

Tables S3-S4.docx **Table S3:** Quantity of *Propithecus diadema* fecal samples per social group. Anka = Ankadivory, the intact forest site. Maha = Mahatsinjo, the disturbed forest site. **Table S4:** Quantity of *Hapalemur griseus* fecal samples per social group. Anka = Ankadivory, the intact forest site. Maha = Mahatsinjo, the disturbed forest site. Tables S3-S4.docx

Table S5.docx **Table S5:** Coinfections of *Cryptosporidium* sp. and *Giardia* sp. a) Humans; b) Pigs; c) Dogs; d) Rats. Table S5.docx
