# Supplementary material for: Cryptosporidium and Giardia prevalence amongst lemurs, humans, domestic animals and black rats in Tsinjoarivo, Madagascar
Source: Heliyon. 2020 Nov 30;6(11):e05604. doi: 10.1016/j.heliyon.2020.e05604 (PMC7711286; doi:10.1016/j.heliyon.2020.e05604)
Supplement: Appendices A-D.docx — A. Recruitment: Information Script B. Informed Consent Documents C. Materials: Questionnaire/Data to be collected D. Debriefing Information: Script for Human Participants with Positive (Infected) Test Results for Cryptosporidium sp. and/or Giardia sp. [file mmc1.docx]

**APPENDIX A**

**RECRUITMENT: INFORMATION SCRIPT**

**Recruitment: Information Script**

**Introduction**

I am ____________________, I am working with Laurie Spencer, a graduate student, at Northern Illinois University. I am helping to conduct research on intestinal parasites in this area, specifically *Cryptosporidium* sp. and *Giardia* sp. I would like to give you some information on this research and invite you to participate in the study. Before you decide to participate, you can talk to anyone you feel comfortable with about the research. Please also ask me to stop as we go through the information if there are words or content that you do not understand. If you have questions later, you can ask me or another researcher.

**Purpose of the research**

*Cryptosporidium* sp. and *Giardia* sp. are common intestinal parasites that can cause illness and can be potentially transmitted between humans and animals. This study will test for the presence of *Cryptosporidium* sp. and *Giardia* sp. in the humans, lemurs, domestic mammals (specifically cattle, pigs and dogs), and rats that inhabit Tsinjoarivo, Madagascar. Fecal samples will be collected from each of these species and will be tested. This study will allow for a better understanding of the *Cryptosporidium* sp. and *Giardia* sp.infections in Tsinjoarivo, Madagascar, and of the potential for parasite transmission amongst humans, lemurs, domestic mammals, and rats, especially as habitat overlap increases.

**Type of Research Intervention**

If you agree to participate, this research will involve getting samples of your stool or your child’s stool. The amount of time required should be less than 10 minutes.

**Participant Selection**

You are being invited to participate in this research because we would like to sample adults and children of your village, since you live adjacent to a forest where illnesses may be transmitted between people, domestic mammals, rats and lemurs, and because incidence of *Cryptosporidium* sp. and *Giardia* sp. infections have been found around Ranomafana National Park not far from this area.

**Voluntary Participation**

Your participation in this research is entirely voluntary. You can choose to participate, or you can choose not to participate. There are no consequences if you choose not to participate. If you choose to participate and then change your mind, you can withdraw from the study at any time.

**Procedures**

We are asking you to help us learn more about *Cryptosporidium* sp. and *Giardia* sp. transmission in your community. We are inviting you to participate in this research project. If you accept, you will be asked for a sample of your stool, or your child’s stool. If I am the owner of domestic animals, cattle, pigs and/or dogs, I will be asked for permission for the researcher(s) to observe my animals for fecal elimination and then collect a stool sample from the ground.

**Stool Collection**

We will give you a sterile tube and instruct you on the procedure for collecting a stool sample properly. If we are asking for your child’s sample, you may assist your child in collecting the sample. The tube with the sample can be returned to the primary researcher or research assistant the following day once you have deposited the sample. These samples will be examined microscopically for the presence of *Cryptosporidium* sp. and *Giardia* sp. You will be given a sample container with a unique sample identification number on it, disposable gloves, biohazard ziplock bag to place the filled sample container, a disposable wooden tongue depressor, and a disposable disinfectant wipe. You will need to collect a portion of fecal material during elimination by using a disposable wooden tongue depressor while wearing the disposable gloves and place the fecal material in the sample container provided and close the container. Then the disposable gloves can be removed, and hands can be sanitized with the disinfectant wipe. The gloves, tongue depressor, and used sanitizing wipe can be placed in a second plastic bag which will be collected by the researcher(s). The filled sample container will be held in the ziplock bag until the researcher(s) collect the sample the next day.

**Risks and Discomforts**

There is a potential for infection by handling your own or your child’s stool sample. This risk will be mitigated by following the collection procedure which includes wearing disposable gloves and sanitizing hands after collection of the stool sample. There is no foreseeable discomfort associated with giving a stool sample.

**Benefits**

Your participation in this study may help us determine which parasites are infecting your community, which may help your community manage illness and its contact with domestic animals and wildlife. Also, if you agree to participate and you or your child is found to have a *Cryptosporidium* sp. or *Giardia* sp. infection, you will be notified of the test results. Also, all participants will be provided multi-vitamins and soap to promote health and hygiene for the prevention and/or recovery from an infection.

**Confidentiality and Results Sharing**

During this study, we will record your name, your age, your sex, where you live, and infection status. Your name and where you live will only be used to contact you, in case you are found to be infected with *Cryptosporidium* sp. or *Giardia* sp. However, we may publish your age, sex, and infection status in scientific literature, and talk about the results we find at scientific gatherings so that people can learn from the research. We may also share your age, sex, and infection status with other researchers or scientists interested in this topic. All files will be kept confidential, and no one in your community will be informed about your participation in this study or its results. Your individual identity will be kept confidential.

**Right to Refuse or Withdraw**

You do not have to take part in this research, and you may withdraw yourself and/or your child whenever you choose. Choosing to participate or choosing not to participate will not affect any foreseeable aspects of your life in any way.

**Who to Contact**

If you have any comments, questions or concerns, you can voice them now or later when your sample is collected. Additionally, you may contact Laurie Spencer (graduate student) or Mitchell Irwin (faculty advisor) at the field research site in Tsinjoarivo, Madagascar in person since no telephone service will be available. If you have any questions or wish to withdraw from this study after the research team has left Madagascar, you can contact Edmond Razanadrakoto in person, who is the local coordinator for the Sadabe organization. Mr. Razanadrakoto can direct your question or comment to the research team or to Research Compliance Office at Northern Illinois University.

**APPENDIX B**

**INFORMED CONSENT DOCUMENTS**

**For Adult Participants (18 Years of Age or Older**)

I agree to participate in the research project titled *Cryptosporidium sp. and Giardia sp. Prevalence amongst Lemurs, Humans, Domestic Animals and Black Rats in Tsinjoarivo, Madagascar* being conducted by Laurie Spencer, a graduate student, at Northern Illinois University.

I have been informed that the purpose of the study is to investigate *Cryptosporidium* sp. and *Giardia* sp., which are common intestinal parasites that can cause illness and can be potentially transmitted between humans and animals. This study will test for the presence of *Cryptosporidium* sp. and *Giardia* sp. in the humans, domestic mammals, rats and lemurs that inhabit Tsinjoarivo, Madagascar. Fecal samples will be collected from each of these species and will be tested. This study will allow for a better understanding of the *Cryptosporidium* sp. and *Giardia* sp. infections in Tsinjoarivo, Madagascar, and of the potential for parasite transmission amongst humans, lemurs, domestic mammals, and rats, especially as habitat overlap increases.

I understand that if I agree to participate in this study, I will be asked to do the following: Collect samples of my stool and provide to the researcher(s). The amount of time required should be less than 10 minutes. If I am the owner of domestic mammals, cattle, pigs and/or dogs, I will be asked to give permission for the researcher(s) to observe my animals for fecal elimination and then collect their stool samples from the ground.

I am aware that my participation is voluntary and may be withdrawn at any time without penalty or prejudice, and that if I have any additional questions concerning this study, I may contact Laurie Spencer (graduate student) or Mitchell Irwin (faculty advisor) at the field research site in Tsinjoarivo, Madagascar in person since no telephone service will be available. I am also aware that if I have any questions or wish to withdraw from this study after the research team has left Madagascar, I can contact Edmond Razanadrakoto in person, who is the local coordinator for the Sadabe organization. Mr. Razanadrakoto can direct my question or comment to the research team or to Research Compliance Office at Northern Illinois University.

I understand that the intended benefits of this study include helping to determine which parasites are infecting my community, which may help my community to manage illness and its contact with domestic mammals and wildlife. If I am found to have a *Cryptosporidium* sp. or *Giardia* sp. infection, I will be notified of the test results. Also, all participants will be provided multi-vitamins and soap to promote health and hygiene for the prevention and/or recovery from an infection.

I have been informed that potential risks and/or discomforts I could experience during this study include the potential for infection by handling of my own stool sample. This risk will be mitigated by following the collection procedure which includes wearing disposable gloves and sanitizing hands after stool sample collection. There are no foreseeable discomforts associated with giving a stool sample. I understand that all information gathered during this experiment will be kept confidential by the researcher(s) who will keep all forms, data sheets and sample containers in their possession. All files will be kept confidential, and no one in my community will be informed about my participation in this study or its results. My individual identity will be kept confidential. During this study my name, age, sex, and where I live (village name), household demographics, frequency of direct contact with animals and with entering the forest, gastrointestinal symptoms, and infection status will be recorded. My name will only be used to contact me, in case I am found to be infected with *Cryptosporidium* sp. or *Giardia* sp. However, all other recorded information may be published in scientific literature, and results may be talked about at scientific gatherings so that people can learn from the research. This information may be shared with other researchers or scientists interested in this topic. In the event I experience a research-related medical emergency or an adverse reaction, please immediately contact Laurie Spencer (graduate student) or Mitchell Irwin (faculty member) at the field research site in Tsinjoarivo, Madagascar in person.

I realize that Northern Illinois University policy does not provide for compensation for, nor does the University carry insurance to cover injury or illness incurred as a result of participation in University sponsored research projects.

I understand that my consent to participate in this project does not constitute a waiver of any legal rights or redress I might have as a result of my participation, and I acknowledge that I have received a copy of this consent form.

Below, check all that apply:

I agree to participate in this study.

I give permission to the researcher(s) to include my animals (cattle, pigs and/or dogs) in this study.

______________________________________________________________________________

Printed Name and Signature of Subject Date

*If Illiterate*:

I have witnessed the accurate reading of the consent form to the potential participant, and the individual has had the opportunity to ask questions. I confirm that the individual has given consent freely.

______________________________________________________________________________

Printed Name and Signature of Witness Date

**Permission Form for Parents/Guardians of Minors (Under 18 Years of Age)**

Your child/ward is invited to participate in a research study titled *Cryptosporidium sp. and Giardia sp. Prevalence amongst Lemurs, Humans, Domestic Animals and Black Rats in Tsinjoarivo, Madagascar* being conducted by Laurie Spencer, a graduate student at Northern Illinois University.

The purpose of the study is to investigate *Cryptosporidium* sp. and *Giardia* sp., which are common intestinal parasites that can cause illness and can be potentially transmitted between humans and animals. This study will test for the presence of *Cryptosporidium* sp. and *Giardia* sp. in the humans, domestic mammals, rats and lemurs that inhabit Tsinjoarivo, Madagascar. Fecal samples will be collected from each of these species and will be tested. This study will allow for a better understanding of the *Cryptosporidium* sp. and *Giardia* sp. infections in Tsinjoarivo, Madagascar, and of the potential for parasite transmission amongst humans, lemurs, domestic mammals, and rats, especially as habitat overlap increases.

Your child's/ward's participation in this study will last approximately 10 minutes. He or she will be asked to collect samples of his or her stool, with parental/guardian assistance if necessary, and provide to the researcher(s).

The following are the foreseeable risks and/or discomforts your child/ward could potentially experience during this study: There is the potential for infection by handling of my child’s/ward’s stool sample. This risk will be mitigated by following the collection procedure which includes wearing disposable gloves and sanitizing hands after stool sample collection. There are no foreseeable discomforts associated with giving a stool sample.

The benefit(s) your child/ward may personally receive from participating in this study include helping to determine which parasites are infecting his or her community, which may help the community to manage illness and its contact with domestic animals and wildlife. If he or she is found to have a *Cryptosporidium* sp. or *Giardia* sp. infection, I as parent/guardian will be notified of the test results. Also, all participants will be provided multi-vitamins and soap to promote health and hygiene for the prevention and/or recovery from an infection.

In the event of a research-related medical emergency or if your child/ward should experience an adverse reaction, please immediately contact Laurie Spencer (graduate student) or Mitchell Irwin (faculty member) at the field research site in Tsinjoarivo, Madagascar in person since no telephone service will be available.

Although Northern Illinois policy does not provide for compensation for treatment of any injuries that may result from participation in research activities, this should not be construed as a waiver of any legal rights or redress you or your child/ward might have as a result of participation in this study.

Information obtained during this study may be published in scientific journals or presented at scientific meetings, but that any information which could identify your child/ward will be kept strictly confidential. All information gathered during this experiment will be kept confidential by the researcher(s) keeping all forms, data sheets and sample containers in their possession. All files will be kept confidential, and no one in your community will be informed about your child’s/ward’s participation in this study or its results. Your child’s/ward’s individual identity will be kept confidential. During this study your child’s/ward’s name, age, sex, and where he/she lives (village name), household demographics, frequency of direct contact with animals and with entering the forest, gastrointestinal symptoms, and infection status will be recorded. Your child’s/ward’s name will only be used for contact purposes in case he/she is found to be infected with *Cryptosporidium* sp. or *Giardia* sp. However, all other recorded information may be published in scientific literature, and results may be talked about at scientific gatherings so that people can learn from the research. This information may be shared with other researchers or scientists interested in this topic.

Participation in this study is voluntary. Your decision whether or not to allow your child/ward, as well as his or her assent to participate will not negatively affect you or your child/ward. Your child/ward will be asked to indicate individual assent to be involved immediately prior to participation and will be free to withdraw from participation at any time without penalty or prejudice.

Any questions about the study should be addressed to Laurie Spencer (graduate student) or Mitchell Irwin (faculty member) at the field research site in Tsinjoarivo, Madagascar in person since no telephone service will be available. If you have any questions or wish to withdraw your child/ward from this study after the research team has left Madagascar, you can contact Edmond Razanadrakoto in person, who is the local coordinator for the Sadabe organization. Mr. Razanadrakoto can direct your question or comment to the research team or to Research Compliance Office at Northern Illinois University.

I agree to allow my child/ward to participate in this research study and acknowledge that I have received a copy of this consent form.

_________________________________________________________________________

Printed Name of Child

_________________________________________________________________________

Printed Name and Signature of Parent/Guardian Date

*If Illiterate*:

I have witnessed the accurate reading of the consent form to the potential participant, and the individual has had the opportunity to ask questions. I confirm that the individual has given consent freely.

_________________________________________________________________________

Printed Name and Signature of Witness Date

**Assent Script for Minors (5 through 17 Years of Age)**

**What is a research study?**

Research studies help us learn new things. We can test new ideas. First, we ask a question. Then we try to find the answer.

**Important things to know…**

- You get to decide if you want to take part in this study.
- You can say ‘No’ or you can say ‘Yes’.
- No one will be upset if you say ‘No’.
- If you say ‘Yes’, you can always say ‘No’ later.
- You can say ‘No’ at any time.
- You can ask any questions at any time to any of the researchers.

**Why are we doing this research?**

We are doing this study to learn more about things that can make people and animals sick. With more information, we can try to find ways to help keep people and animals healthy.

**What would happen if I join this research?**

If you decide to be in the research, we would ask you to do the following:

- - Stool sample: We would ask you to collect a sample of your stool, either by yourself or with the help of your parent/guardian. Then you would give the sample to one of the researchers of this study. This should take less than 10 minutes of your time.
  - Talking: One of the researchers would ask you a few questions. Then you would say your answers out loud.

**Could bad things happen if I join this research?**

Touching your stool may make you uncomfortable or the questions might be embarrassing to answer. We will try to make sure that you feel comfortable during this study.

**Do you want to be a part of this study?**

**Confidentiality Statement for Research Assistants**

I _____________________, as a research assistant working with Laurie Spencer, a graduate student, at Northern Illinois University (NIU), agree to help conduct research on the intestinal parasites *Cryptosporidium* sp. and *Giardia* sp. I understand that part of this research involves obtaining personal information about people in the local community, like their names, where they live, and their *Cryptosporidium* sp. and *Giardia* sp. test results. I agree to not share or discuss any participant's test results or personal identification information with any other people besides the primary researcher Laurie Spencer, or Dr. Irwin (NIU faculty advisor).

Printed Name and Signature of Research Assistant Date

**APPENDIX C**

**MATERIALS: QUESTIONNAIRE/DATA TO BE COLLECTED**

**Materials: Questionnaire/Data to be collected**

1. Sample Collection Number:
2. Participant’s full name:
3. If under age 18, name of parent/guardian:
4. Age:
5. Sex:
6. Village name:
7. Has the participant experienced any gastrointestinal symptoms (i.e. diarrhea) within the last month (if yes, explain symptoms) : ________________________________

___________________________________________________________________

1. Number of people in household (total):
   1. Number of adults:
   2. Number of minors:
2. Number of domestic mammals in household (total):
   1. Number of cattle:
   2. Number of pigs:
   3. Number of dogs:
3. Frequency of direct contact with domestic mammals (check appropriate box):

None

Once

2 to 4 times

Once per week

Every day

Don’t know

Other: _____________________________________________________

1. Frequency of entering the forest within the last month (check appropriate box):

None

Once

2 to 4 times

Once per week

Every day

Don’t know

Other: _____________________________________________________

1. Frequency of direct contact with rats within the last month (check appropriate box):

None

Once

2 to 4 times

Once per week

Every day

Don’t know

Other: _____________________________________________________

1. Frequency of direct contact with lemurs within the last month (check appropriate box):

None

Once

2 to 4 times

Once per week

Every day

Don’t know

Other: _____________________________________________________

1. Infection Status (positive or negative for *Cryptosporidium* sp. or *Giardia* sp.):

**APPENDIX D**

**DEBRIEFING INFORMATION – SCRIPT FOR HUMAN PARTICIPANTS WITH POSITIVE (INFECTED) TEST RESULTS FOR *CRYPTOSPORIDIUM* AND *GIARDIA***

**Debriefing Information – Script for Human Participants with Positive (Infected) Test Results for *Cryptosporidium* sp. and *Giardia* sp.**

I am ____________________, I am working with Laurie Spencer, graduate student at Northern Illinois University, to conduct research on the intestinal parasites *Cryptosporidium* sp. and *Giardia* sp. Testing of your (or your child’s) stool sample showed that you are positive (infected) for *Cryptosporidium* sp. or *Giardia* sp. I would like to give you some recommendations for actions to take.

1. Wash hands frequently, especially when in contact with fecal matter of any kind, when in contact with animals or with soil.
2. Since *Cryptosporidium* sp. and *Giardia* sp. infections can cause diarrhea, drink lots of clean water.
3. Always boil water before drinking.
4. Most healthy people do not need specific medical treatment for *Cryptosporidium* sp. and *Giardia* sp., but if symptoms are severe or persist for more than one week, seek medical attention at a health clinic. No medications are known to be effective for treating *Cryptosporidium* sp. infections. *Giardia* sp. infections can be treated with certain antibiotics by a healthcare professional.
