# Supplementary figures and images for: Cryptosporidium and Giardia prevalence amongst lemurs, humans, domestic animals and black rats in Tsinjoarivo, Madagascar
Source: Heliyon. 2020 Nov 30;6(11):e05604. doi: 10.1016/j.heliyon.2020.e05604 (PMC7711286; doi:10.1016/j.heliyon.2020.e05604)

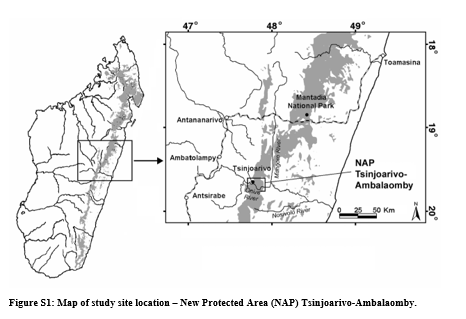

Supplement: Figure S1.docx — Figure S1: Map of study site location – New Protected Area (NAP) Tsinjoarivo-Ambalaomby. [file mmc2.docx]

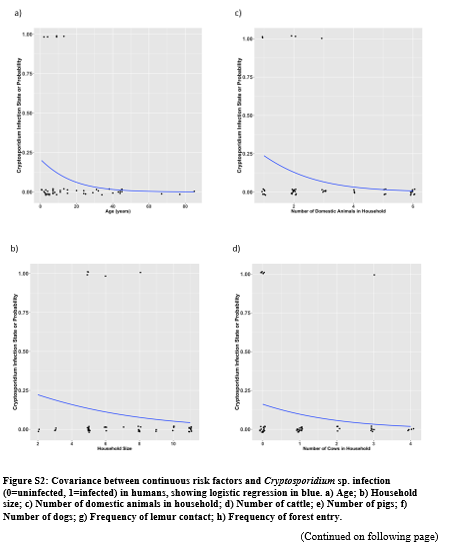


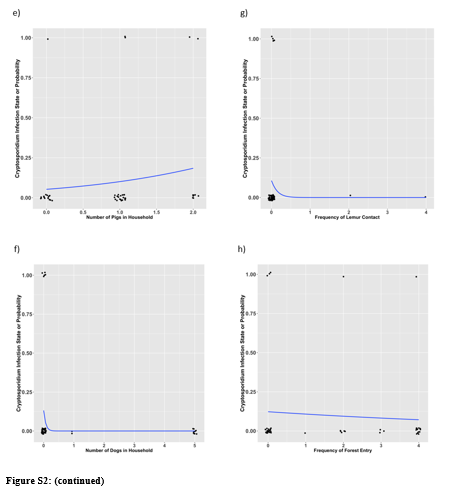


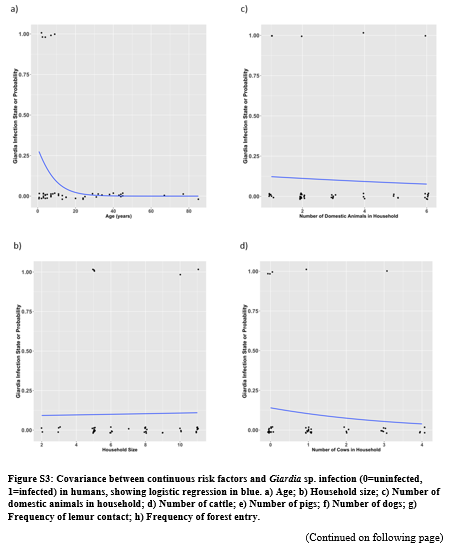


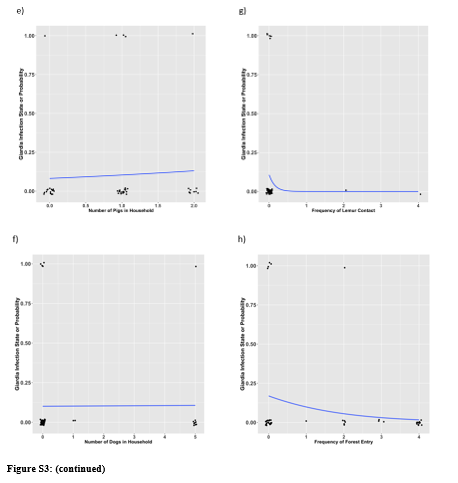


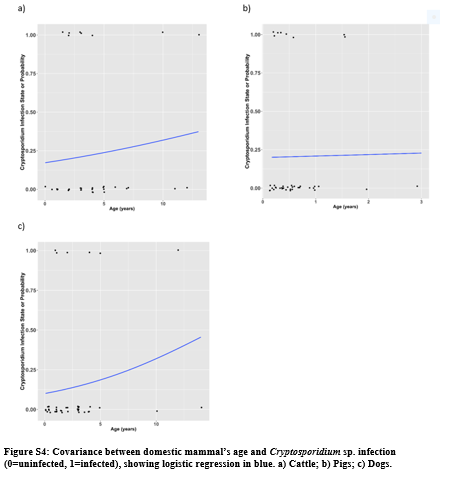


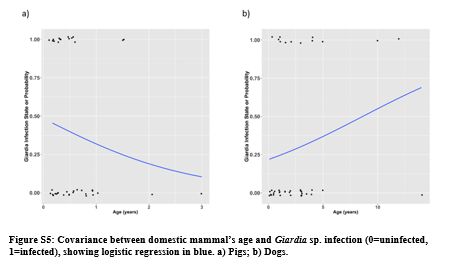


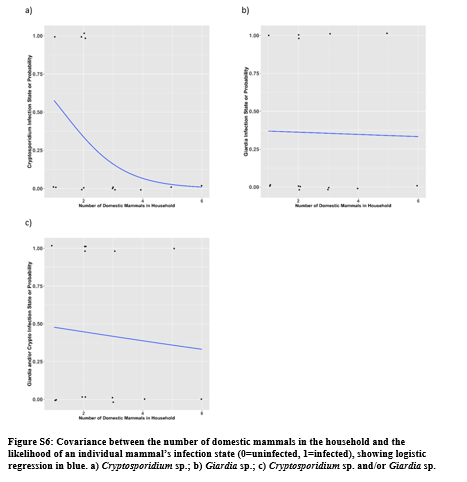

Supplement: Figures S2-S6.docx — Figure S2: Covariance between continuous risk factors and Cryptosporidium sp. infection (0=uninfected, 1=infected) in humans, showing logistic regression in blue. a) Age; b) Household size; c) Number of domestic animals in household; d) Number of cattle; e) Number of pigs; f) Number of dogs; g) Frequency of lemur contact; h) Frequency of forest entry. Figure S3: Covariance between continuous risk factors and Giardia sp. infection (0=uninfected, 1=infected) in humans, showing logistic regression in blue. a) Age; b) Household size; c) Number of domestic animals in household; d) Number of cattle; e) Number of pigs; f) Number of dogs; g) Frequency of lemur contact; h) Frequency of forest entry. Figure S4: Covariance between domestic mammal’s age and Cryptosporidium sp. infection (0=uninfected, 1=infected), showing logistic regression in blue. a) Cattle; b) Pigs; c) Dogs. Figure S5: Covariance between domestic mammal’s age and Giardia sp. infection (0=uninfected, 1=infected), showing logistic regression in blue. a) Pigs; b) Dogs. Figure S6: Covariance between the number of domestic mammals in the household and the likelihood of an individual mammal’s infection state (0=uninfected, 1=infected), showing logistic regression in blue. a) Cryptosporidium sp.; b) Giardia sp.; c) Cryptosporidium sp. and/or Giardia sp. [file mmc3.docx]

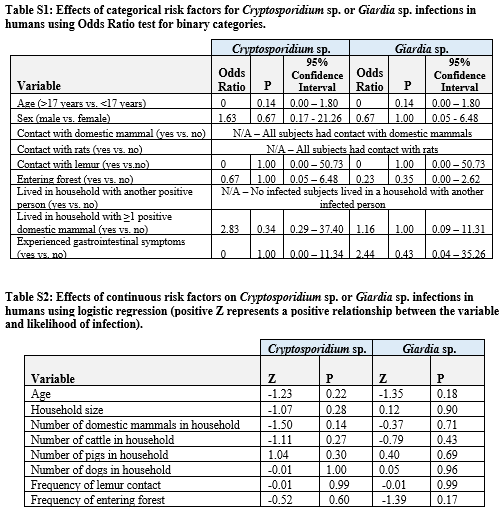

Supplement: Tables S1-S2.docx — Table S1: Effects of categorical risk factors for Cryptosporidium sp. or Giardia sp. infections in humans using Odds Ratio test for binary categories. Table S2: Effects of continuous risk factors on Cryptosporidium sp. or Giardia sp. infections in humans using logistic regression (positive Z represents a positive relationship between the variable and likelihood of infection). [file mmc4.docx]

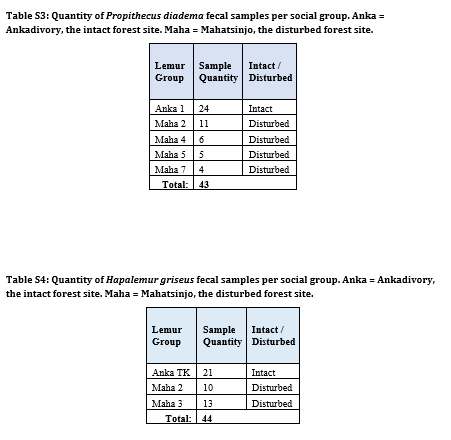

Supplement: Tables S3-S4.docx — Table S3: Quantity of Propithecus diadema fecal samples per social group. Anka = Ankadivory, the intact forest site. Maha = Mahatsinjo, the disturbed forest site. Table S4: Quantity of Hapalemur griseus fecal samples per social group. Anka = Ankadivory, the intact forest site. Maha = Mahatsinjo, the disturbed forest site. [file mmc5.docx]

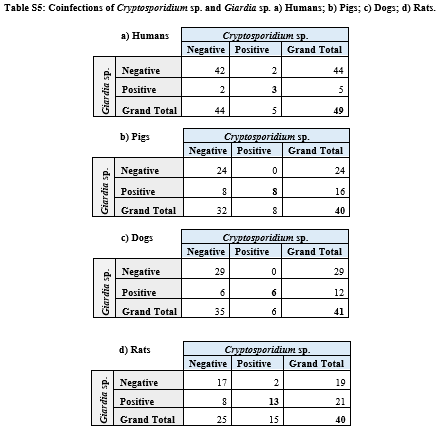

Supplement: Table S5.docx — Table S5: Coinfections of Cryptosporidium sp. and Giardia sp. a) Humans; b) Pigs; c) Dogs; d) Rats. [file mmc6.docx]
